# Genomic Analysis of Individual Differences in Ethanol Drinking: Evidence for Non-Genetic Factors in C57BL/6 Mice

**DOI:** 10.1371/journal.pone.0021100

**Published:** 2011-06-16

**Authors:** Jennifer T. Wolstenholme, Jon A. Warner, Maria I. Capparuccini, Kellie J. Archer, Keith L. Shelton, Michael F. Miles

**Affiliations:** 1 Department of Pharmacology and Toxicology, Virginia Commonwealth University, Richmond, Virginia, United States of America; 2 Department of Biostatistics, Virginia Commonwealth University, Richmond, Virginia, United States of America; 3 Department of Neurology, Virginia Commonwealth University, Richmond, Virginia, United States of America; University of Queensland, Australia

## Abstract

Genetic analysis of factors affecting risk to develop excessive ethanol drinking has been extensively studied in humans and animal models for over 20 years. However, little progress has been made in determining molecular mechanisms underlying environmental or non-genetic events contributing to variation in ethanol drinking. Here, we identify persistent and substantial variation in ethanol drinking behavior within an inbred mouse strain and utilize this model to identify gene networks influencing such “non-genetic” variation in ethanol intake. C57BL/6NCrl mice showed persistent inter-individual variation of ethanol intake in a two-bottle choice paradigm over a three-week period, ranging from less than 1 g/kg to over 14 g/kg ethanol in an 18 h interval. Differences in sweet or bitter taste susceptibility or litter effects did not appreciably correlate with ethanol intake variation. Whole genome microarray expression analysis in nucleus accumbens, prefrontal cortex and ventral midbrain region of individual animals identified gene expression patterns correlated with ethanol intake. Results included several gene networks previously implicated in ethanol behaviors, such as glutamate signaling, BDNF and genes involved in synaptic vesicle function. Additionally, genes functioning in epigenetic chromatin or DNA modifications such as acetylation and/or methylation also had expression patterns correlated with ethanol intake. In verification for the significance of the expression findings, we found that a histone deacetylase inhibitor, trichostatin A, caused an increase in 2-bottle ethanol intake. Our results thus implicate specific brain regional gene networks, including chromatin modification factors, as potentially important mechanisms underlying individual variation in ethanol intake.

## Introduction

Over 121 million Americans drink alcohol, while less than 10% of the population drinks excessively [1], [2]. In 2000, alcohol consumption and alcoholism were responsible for 3.5% of all deaths in the United States and cost over $185 billion annually [3]. These facts highlight the importance of identifying those factors that may influence the variability in drinking behaviors. Extensive studies in humans have suggested that genetic factors account for about 40–60% of the risk for alcoholism [4], [5], [6], [7]. Work in humans and animal models over the last 20 years has documented genetic intervals [8], [9], [10] or individual genes [11], [12] contributing to variation in behavioral responses to ethanol.

Despite such progress on identifying genetic influences in alcoholism, little progress at the molecular level has revealed mechanisms that mediate environmental influences on ethanol behaviors or alcoholism. It is well documented that environmental influences such as stress or exposure to conditional stimuli can modify ethanol drinking or cause recidivism in abstinent alcoholics. Understanding the molecular mechanisms underlying such environmental influences on ethanol behaviors would augment the genetic progress mentioned above.

C57BL/6 (B6) inbred mice have been widely used as a model for studying alcohol abuse related behaviors and the genetic basis of alcohol abuse since these mice voluntarily consume large volumes of unadulterated ethanol [10], [13], [14], [15]. However, a number of prior studies have documented remarkable degrees of stable, individual variation in 2-bottle choice drinking behavior in rodents including several studies that have shown individual variation can occur within a single inbred strain including C57 substrains C57BL/6J [16] and C57BL/10 [17], [18]. This eliminates factors such as genetic differences in taste or ethanol reward as causal for the variation in drinking behavior. Studies in C57BL/6J mice suggest that non-genetic persistent individual differences in drinking behavior are the major source of variance in ethanol drinking in these animals, outweighing substantial environmental challenges such as diet [16]. Using such a model, where genetic factors are strictly controlled, offers considerable power for studying molecular mechanisms of environmental modulation of ethanol drinking behavior.

Here, we demonstrate a remarkable degree of individual variation in ethanol drinking behavior across individual mice from the C57BL/6NCrl inbred line. We have performed whole genome expression profiling in individual mice to finely dissect molecular factors underlying this individual variation in ethanol drinking behavior. We hypothesized that an as yet unidentified non-genetic factor has caused long-lasting brain signaling alterations that influence ethanol preference and intake in these mice. By characterizing gene networks differentially expressed between ethanol preferring and avoiding mice, we have identified putative epigenetic mechanisms such as alterations in chromatin acetylation that may regulate gene transcription and influence drinking patterns. We expect that these studies may contribute to the identification of novel targets for pharmacotherapy in alcoholism.

## Methods

### Ethics Statement

All procedures were approved by Virginia Commonwealth University Institutional Animal Care and Use Committee under protocol numbers AM10332 and AM10139, and followed the NIH Guide for the Care and Use of Laboratory Animals (NIH Publications No. 80–23, 1996).

### Animals

Male C57BL/6NCrl mice (age 42–49 days) from Charles River Laboratories (Wilmington, MA) were habituated to the vivarium (5 mice/cage) for 1 week followed by individual housing for 1 week prior to beginning drinking experiments. Cages and bedding (Harlan Sani-chips, #7090A, Harlan, Teklad, Madison, WI) were changed weekly. Mice were housed in a temperature and light controlled room (12∶12 h cycle, lights on at 0600) with free access to standard chow (Harlan Teklad #7912, Madison, WI) and water.

### Two-bottle choice drinking

Experiment 1: Voluntary two-bottle choice drinking was performed as described previously [19]. Two bottles containing 10%(w/v) ethanol (Aaper Alcohol and Chemical Co. Shelbyville, KY) or tap water were placed into the home cage at the beginning of the dark cycle. Tube position was varied every two days (L, L, R, R). Drinking sessions lasted 18 hours/day followed by 6 hours access to water only. Mice had four consecutive drinking sessions followed by four days of abstinence repeated four times to give 16 total drinking sessions. Tissues from prefrontal cortex (PFC), nucleus accumbens (NAc) and the ventral midbrain region (VMB; including the ventral tegmental area) were harvested 6 days after the last drinking session for microarray analysis as previously described [20]. Tissue was stored at −80°C until RNA isolation. Experiment 2 was conducted in an identical manner (n-21 mice) except tissue was harvested for Western blotting as described below.

Experiment 3: For studies on ethanol preference in littermates, two cohorts of male B6 littermates were purchased as littermates from Charles River. Breeding at Charles River facility used two females paired to one male. Offspring were weaned at day 21, shipped to our vivarium at day 35 and remained housed as littermates until the beginning of the study. 10 litters were represented with 3–5 males per litter (n = 36 total). Mice were individually housed for 7 days then presented with 10%(w/v) ethanol for 18 h/day in a two-bottle choice paradigm for 14 days.

### Taste Discrimination

Experiment 4: Taste preference for a bitter or sweet solution was measured using quinine or saccharin vs. water in a two-bottle choice paradigm. Mice (n = 16) were tested for ethanol preference for 14 days as in experiment 1 but without deprivation periods, and then allowed to rest for 7 days with only water and food available. Half the mice were given two bottles containing either a 0.1 mM quinine solution or tap water and the others given a choice between 0.033% saccharin/water. Bottles were alternated every other day. Consumption of quinine/water or saccharin/water was measured daily for 3 days (18 h/day) after which the other tastant was offered for 3 days in a counterbalanced design.

### HDAC Inhibitor Studies

Experiment 5: 18 male C57BL/6NCrl mice, acclimated to a reverse-light cycle, were used to test effects of trichostatin A (TSA, a class I and II HDAC inhibitor) on ethanol drinking. Voluntary ethanol drinking was initiated as before except these mice had access 24 hours/day, changed at 1200 h. Following 7 days baseline ethanol access, mice were divided into two groups balanced for baseline intake. Mice in the TSA group (n = 9) were injected with 2 mg/kg TSA i.p. (dissolved in 1∶5 DMSO:saline) for 5 days. Control mice (n = 9) were injected with vehicle. Mice had continuous ethanol access during the entire study (39 days total).

Experiment 6: Western blotting for acetyl-Histone-H4 was used to verify CNS activity of i.p. TSA. C57BL/6NCrl mice (n = 12) received a single i.p. injection of 2 mg/kg TSA or vehicle. After 24 hours, nucleus accumbens was dissected for Western blotting as described below.

The effect of TSA on ethanol metabolism was assayed in a separate group of mice (Experiment 7). Male C57BL/6NCrl mice (n = 4−5/group; 37 mice total) received 2 mg/kg TSA or vehicle for 5 consecutive days. On day 6, all mice were injected with 2 mg/kg ethanol. Trunk blood was harvested from mice at 10, 60, 120 and 180 minutes following ethanol administration. Whole blood samples (20 µl) were placed into 20-ml headspace vials with 960 µl water and 20 µl 1-propanol internal standard. Samples were tested for ethanol concentration using a Hewlett Packard 5890A gas chromatograph equipped with a flame ionization detector, 2 meter 5% Carbowax 20M 80/120 mesh packed column (Restek, Bellefonte, PA) and CTC Combi-Pal headspace autosampler. Data were acquired by Clarity software (Apex data systems, Prague, CZ) and analyzed by linear regression with no weighting. A 7-point calibration curve preceded the analysis and quality control ethanol standards were interspersed with each set of samples. Up to 3 replicates were analyzed from each animal and averaged if sufficient blood was collected.

### RNA isolation and microarray hybridization

Total RNA was extracted from PFC, NAc and VMB from individual mice in STAT 60 reagent (Tel-Test, Friendswood, TX) according to the manufacturer's protocol. RNA concentration and quality was assessed by Experion automated electrophoresis (BioRad, Hercules, CA). Total RNA (2 *µ*g) was transcribed into double-stranded cDNA using the One-cycle Targeting and Control Reagent kit (Affymetrix, Santa Clara, CA). Biotin-labeled cRNA was synthesized from cDNA, purified and fragmented according to manufacturer's instructions.

Labeled cRNA from individual animals (n = 19) was hybridized to a single microarray for each brain region (n = 57 total microarrays). Samples were analyzed on oligonucleotide arrays (Mouse Genome 430A 2.0 array) containing >22,000 well-characterized genes and expressed sequence tags. Array hybridization and scanning were performed exactly according to manufacturer's protocols (Affymetrix).

### Microarray data analysis

Microarray data were processed using GeneChip Operating Software v4.1 (GCOS, Affymetrix) and normalized to a mean total hybridization intensity of 190. All raw microarray data is deposited at the Gene Expression Omnibus repository (GEO) under accession number GSE26506. All microarray data is MIAME compliant. Array quality was assessed by accepting arrays with scaling factor <3, 3′–5′-actin ratio <2, and by examining linearity and inter-chip correlations of intensity values. Arrays determined to be acceptable (57/58) were further analyzed using the Robust Multichip Average (RMA) low-level analysis algorithm to summarize probeset expression data [21]. Probesets with RMA expression values <4.5 consistently across all microarrays were filtered to reduce variance from low expressing genes. To identify gene expression correlated with ethanol drinking behavior across individual mice, RMA values for each brain region were correlated [22] to a drinking scale calculated from each mouse's ethanol intake averaged over the last 8 drinking days using the template matching tool in T-Mev (TIGR Multiple expression viewer [23]). P-values from the template matching analysis were then used in estimating the false discovery rate (FDR) using the q value method [24] in the R programming environment [25]. Probesets were considered significant using a FDR of 1%. Significant probesets were further studied for functional gene sets by hierarchical clustering (T-MeV, average linkage) and bioinformatics analyses as below.

The Expression Analysis Systematic Explorer (EASE version 1.21) [26] nonbiased annotation analysis tool was used to identify biological themes among gene expression profiles and to group genes into functional classifications. EASE results were filtered to remove categories with more than 250 members and EASE scores of >0.05. Redundant categories with the same gene members were removed to yield a single representative category. Additional bioinformatics analysis of gene lists were performed with Ingenuity Pathway Analysis (Ingenuity® Systems, www.ingenuity.com) and Bibliosphere (http://www.genomatix.de). These tools utilize biomedical literature associations to annotate genes with biological functions and cellular components. IPA also generates networks of interrelated genes based on their curated knowledge base.

### Principal Component Analysis

As an alternative to correlating gene expression to the average of the last 8 days of ethanol drinking data, we conducted a principal components analysis to reduce the number of covariates and identify the few principal components that account for a sufficient proportion of the variability and contain almost as much information as the full data set. The first 2 principal components accounted for 0.77 of the total variance. For each brain region, probeset-specific linear models predicting expression as a function of the two independent principal components were fit. An overall F-test was used for calculating P-values for each probe set level linear model. Genes significant at p<0.05 level were selected for further bioinformatics analysis and comparison with results from using average ethanol intake over the last eight days.

### Association with alcohol-preferring and non-preferring mouse models

Genes significantly correlated to ethanol drinking patterns in the present study, using a false discovery rate of 1%, were analyzed for overlap with previously published gene sets having expression significantly different between alcohol-preferring or non-preferring mouse models based on the criteria |*d*|≥0.5 and *q*<0.05 [27]. Genes intersecting between these data sets and the studies performed here were further analyzed using bioinformatics tools as previously described.

### Western blot analysis

Twenty-one C57BL/6NCrl mice (Exp. 2) voluntarily consumed ethanol as described above and brains were harvested six days following the last drinking session. Nucleus accumbens was homogenized in NP40 buffer with protease inhibitors (Roche, Indianapolis, IN). Western blotting was performed as described [20]. RAB3A blots were probed with rabbit anti-RAB3A (Millipore, Bedford, MA) and mouse anti-beta-actin (AbCam, Cambridge, MA) and visualized with HRP (GE Healthcare, Buckinghamshire, UK) and ECL reagent (Amersham Biosciences, Piscataway, NJ). Acetyl-Histone H4 blots were probed with rabbit anti-acH4 (AbCam, Cambridge, MA) and mouse anti-GAPDH (Millipore, Bedford, MA). Images were digitized and protein expression was determined as area under the curve normalized to beta-actin using ImageJ (NIH, Bethesda, MD).

## Results

### Individual Variation in Ethanol Drinking Behaviors

**Figure 1:**
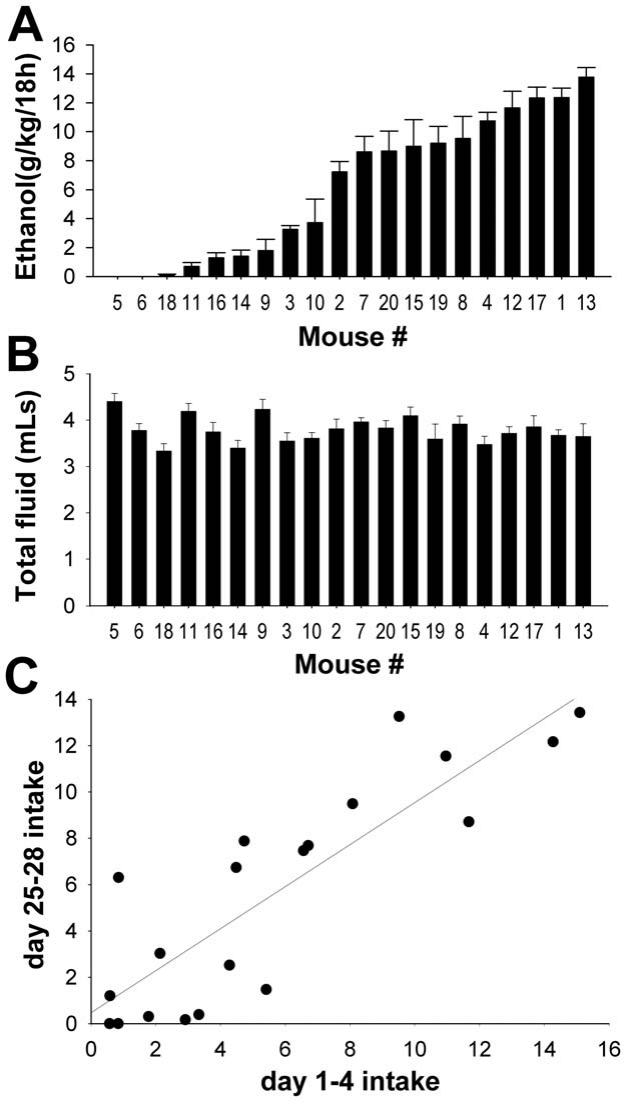
Ethanol drinking in individual C57BL/6NCrl mice. **A.** Ethanol intake expressed in grams per kilogram body weight over 18 hours of ethanol access. Mice show a robust, but persistent variation in ethanol drinking. **B.** Total liquid consumed (ml ethanol + ml water) in 18 h/day. **C.** Scattergram of ethanol drinking on days 1–4 versus days 17–28, correlation R = 0.738, p<0.0002.

C57BL/6 (B6) male mice from Charles River Laboratories consumed a substantial amount of ethanol, 6.47±0.99 g/kg/18 h, in the voluntary two-bottle choice (10% w/v ethanol or water) self-administration paradigm. Interestingly, mice showed a large degree of inter-individual variation in ethanol drinking (Figure 1a), ranging from 0.28±0.14 g/kg/18 h to 14.39±0.47 g/kg/18 h. This corresponds to almost complete ethanol abstinence (ethanol preference, 0.015±0.0074) to very high ethanol preference (0.95±0.035). Ethanol preference was significantly correlated to ethanol intake (R = 0.949, p<0.001 Pearson Correlation) since there were minimal differences in total fluid consumed (Figure 1b). The only mouse with significantly higher fluid consumption had the lowest ethanol intake and preference. The variation in ethanol intake across individual mice was very consistent over the course of the drinking sessions (Figure 1c). Ethanol intake for the first 4 days of drinking was highly correlated with intake over the last 4 days of drinking (R = 0.676, p = 0.0011, day1–4 vs. day 25–28, see Table 1). This stability suggested that most of the observed variance was due to between-subject individual differences rather than random environmental factors.

**Table 1:** Correlation of initial ethanol intake versus subsequent rounds of drinking following deprivation.

	d1-4 intake	d9-12 intake	d17-20 intake	d25-28 intake
d1-4 intake	1.000	0.703	0.772	0.676
d9-12 intake	0.703	1.000	0.850	0.779
d17-20 intake	0.772	0.850	1.000	0.993
d25-28 intake	0.676	0.779	0.993	1.000

To assess whether taste discrimination was contributing to variation in ethanol drinking, we performed an additional experiment (Exp. 4) where mice (n = 16) were assessed for ethanol drinking, followed by studies on preference for quinine (0.1 mM) or saccharin (0.033%). While there was some individual variation in quinine consumption, preference for saccharin (R = 0.142, p = 0.589) or quinine (R = 0.196, p = 0.468) showed no significant correlation to ethanol preference (data not shown). These results argue against sweet or bitter taste as a major contributing factor for the observed individual differences in ethanol preference.

Additionally, we determined whether the observed individual variation in ethanol drinking was due to robust differences between litters (Exp. 3). In two separate cohorts consisting of 10 litters (n = 3–5 males/litter, 36 total), ethanol intake over 14 days of baseline drinking did not differ between litters (F(9,32) = 1.258 p = 0.2967, Table S1). Ethanol preference also did not differ between litters (F(9,32) =  1.629, p = 0.1489, Table S1). The average ethanol intake in the littermate study (6.96±0.53 g/kg/18 h) did not differ significantly from the average ethanol intake in the non-littermate studies (Fig. 1). Additionally, the range of drinkers (0.8 – 10.8 g/kg/18 h) was very similar to that shown in Fig. 1 and was evenly distributed between the litters, suggesting that simple litter effect differences do not greatly contribute to the inter-individual variability of intake in these mice. While these observations argue against litter effects playing a major contribution to the individual variability in ethanol drinking, one cannot fully exclude the possibility that other factors, such as sex composition of the litter may alter hormonal exposure levels during the time when hormones organize the brain and affect an animal's drug response. A much larger study design would be needed to tease out such minor contributions.

### Differential gene expression in ethanol preferring and avoiding mice

We hypothesized that persistent individual variation in ethanol drinking behaviors within an inbred strain might be caused by differential basal gene expression patterns generated by unknown environmental influences. Further, such differential gene expression patterns could be used to identify molecular pathways contributing to individual variation in ethanol drinking. We profiled 3 brain regions in individual mice: nucleus accumbens (NAc), prefrontal cortex (PFC) and ventral midbrain region (VMB). These brain regions were chosen because they are major components of the mesocorticolimbic dopamine reward pathway activated by ethanol and other drugs of abuse [28]. Pair-wise comparisons of microarrays showed gene intensities of individual arrays were highly correlated with the lowest Pearson correlation value being 0.97 (not shown). Each array passed rigorous quality control checks in our laboratory showing that micro-dissected brain regions from individual animals could be reliably analyzed by microarrays without requiring sequential rounds of probe amplification.

To identify molecular factors related to ethanol drinking behaviors, gene expression patterns were first correlated to a drinking template created from the last 8 days of ethanol access following a third round of ethanol deprivation (see Methods). This design was chosen because the mice did not show an ethanol deprivation effect after this time point (Figure S1 and [19]). As we have reported previously, mice showed a diminishing deprivation effect after the first and second abstinence periods that disappeared with the third abstinence. Utilizing multiple rounds of ethanol deprivation enabled assessment of the stable individual ethanol intake while providing a window where tissue could be harvested with animals off ethanol. Correlations of ethanol intake and gene expression were performed separately for each brain region, using a false discovery rate of 1% (see Table S2 for gene lists). The number of genes significantly correlated to ethanol drinking was similar in NAc and PFC with fewer transcripts regulated in the VMB (Figure S2). Not surprisingly, there was little overlap in the identity of significant genes across brain regions (Figure S2). Therefore, gene expression data from each brain region was further analyzed separately.

To identify gene expression correlated with drinking behavior, we also performed a principal component analysis on the daily drinking activity data to reduce the number of covariates, rather than averaging the drinking data over an interval. The first two principal components (PC) accounted for 77% of the total variance. For each brain region, probe set-specific linear models predicting expression as a function of the two independent principal components were fit. The number of transcripts which fit the linear model at a level p<0.05 was 547 in NAc, 670 in PFC and 725 in VMB (Table S3). When these data were intersected with the results from analysis of averaged drinking intake, a highly significant degree of overlap was found between the two results. Of the number of transcript correlating with averaged drinking behavior, overlap with the PC analysis was found for 291 (33%) genes in NAc, 223 in PFC (26%) and 154 in VMB (27%).

### Bioinformatics Analysis of Regional Microarray Data

Gene lists from microarray analyses were analyzed for over-representation of biological functions or gene network relationships using several different tools as described in Methods. As mentioned below, there was a striking similarity between gene lists resulting from analysis of either average drinking data or the PC data, from both PFC and NAc. Since the correlations to average drinking values generated larger gene lists, we focused our analysis on these data and the genes showing overlap with the PC analysis.

#### Nucleus Accumbens

The 889 transcripts from NAc correlating with average drinking values were analyzed by EASE [26] for overrepresentation of functional categories compared with all genes on the Mouse 430Av2 chip (Table S4). Major significant groups include genes associated with synaptic vesicles, protein transport, protein ubiquitination, chromatin modifications and histone deacetylase complex as well as categories related to small GTPase signal transduction, cytoskeletal organization and kinase activity. A majority of the categories were also identified by analysis with Bibliosphere and are bolded in Table S4. The top canonical pathways identified by Ingenuity Pathway Analysis (see below) also mirrored the Gene Ontology results. Phosphoinositol 3 kinase/Akt signaling, ephrin receptor signaling, PDGF signaling, protein ubiquitination, and inositol metabolism were among the significant canonical pathways.

**Figure 2:**
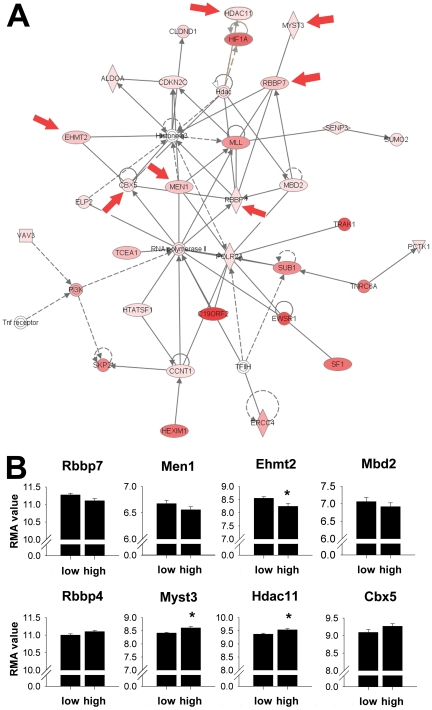
Chromatin modification genes differentially regulated in the nucleus accumbens of ethanol drinking mice. A. Network of genes involved in chromatin modification generated through the use of Ingenuity Pathways Analysis (www.ingenuity.com). Red arrows indicate genes identified in Gene Ontology Biological Process for establishment and/or maintenance of chromatin architecture. Genes significantly correlated to ethanol drinking are colored pink to red based on significance. Solid arrowheads reflect “acts on” interactions while lines without arrow indicate binding interactions only. Solid and dotted lines indicate, respectively, direct vs. indirect interactions. **B**. RMA expression of transcripts in networks involved in chromatin architecture. Low and high refer to the lowest vs. highest quartiles of ethanol drinkers. * p<0.05 by t-test.

Biological functions of our gene lists were further investigated using the curated knowledge base in Ingenuity Pathway Analysis. This tool generates networks of genes with known interactions or biological function. One of the top networks generated through this process is shown in Figure 2. This network included multiple genes related to chromatin modification and regulation of transcription through possible epigenetic mechanisms. Seven of the genes in this network were also identified in the Gene Ontology Biological Process category for establishment and/or maintenance of chromatin architecture and are identified with arrows. Additionally, 5 probesets were identified in the Gene Ontology cellular component for the histone deacetylase complex: *Hdac11* (NM_144919.2), *Rbbp4* (NM_009030.3), *Rbbp7* (NM_009031.3), *Sap18 (NM_009119.3)* and *Suds3* (NM_178622.4). All genes in this network were significantly correlated to average ethanol drinking (Table 2). *Rbbp4* and *Rbbp7*, retinoblastoma-binding proteins, together with HDAC 1 and HDAC2, form the HDAC core which is part of both the NuRD and Sin3a complexes involved in transcriptional repression [29]. We previously showed that acute ethanol treatment increases mRNA levels of *Rbbp4* [20]. *Hdac11*, histone deacetylase complex 11, functions to repress RNA expression by removing acetyl groups from the core histones allowing DNA packaging into dense chromatin structures [30]. Other genes in the network (*Mbd2, Mll1, Men1, Ehmt2*, and *Dnmt1*) are involved in DNA methylation events and work concurrently to repress transcription [31], [32].

**Table 2:** Genes involved in chromatin remodeling identified in nucleus accumbens.

Gene Symbol	Gene Name	Probeset ID	R value	p value	Function	Transcription
***Mbd2***	**methyl-CpG binding domain protein 2**	1425803_a_at	−0.515	1.00E-03	binds methylated DNA	silences
***Men1***	**multiple endocrine neoplasia 1**	1416348_at	−0.480	2.84E-03	methylated Lys4 Histone H3	silences
***Ehmt2***	**euchromatic histone lysine N-methyltransferase 2**	1426888_at	−0.482	2.69E-03	methylates Lys9 Histone H3	silences
*Rbbp7*	retinoblastoma binding protein 7	1415775_at	−0.481	2.75E-03	subunit of core HDAC complex	silences
***Rbbp4***	**retinoblastoma binding protein 4**	1434892_x_at	0.505	1.39E-03	member of NuRD and Sin3A complex	silences
***Myst3***	**MYST histone acetyltransferase 3**	1436315_at	0.650	7.92E-06	acetylated histones	activates
***Hdac11***	**histone deacetylase 11**	1451229_at	0.522	8.34E-04	deacetylates histones	silences
*Cbx5*	chromobox homolog 5 (Drosophila HP1a)	1454636_at	0.512	1.10E-03	binds acetylated histone 3	silences

Gene names in bold were also identified by the principal component analysis.

The relative expression of select genes in this network in the top quartile of high ethanol drinkers (>7 g/kg/18 h) and the bottom quartile of low ethanol drinkers (<2 g/kg/18 h) are summarized in Figure 2B. *Myst3* (NM_001081149), *Hdac11* and *Ehmt2 (*NM_147151*)* were significantly different in high versus low drinkers by t-test at p<0.05. *Myst3*, Myst histone acetyl transferase 3, is a member of a mouse histone acetyl transferase (HAT) complex that increases DNA transcription [33] by acetylating histone tails. Acetylation of histone tails opens up the chromatin structure to allow transcription factors and associated proteins access to the DNA and increase gene transcription. Many genes were in HDAC complexes (*Rbbp4, Rbbp7*), had intrinsic HDAC activity (*Hdac11*) or were involved in methylation of DNA (*Men1* (NM_001168488), *Mbd2* (NM_010773.2), *Mll1* (NM_001081049), *Ehmt2)*. These genes are believed to cause transcriptional silencing by removing acetyl groups from chromatin or increasing DNA methylation.

Further network analysis identified genes involved in synaptic vesicle formation and recycling (see Table 3). Genes involved in dynamin-dependent vesicle recycling (*Ap2a1* (NM_025606), *Ap2a2* (NM_007459), *Ap2m1* (NM_207255), *Dnm1* (NM_144516), *Dnm1l* (NM_028661), *Vamp3* (NM_009498), and *Vamp4* (NM_016796.3)) and synaptic vesicle biogenesis (*Sh3gl2* (NM_019535), *Sh3glb1* (NM_175141)) were generally positively correlated to ethanol intake. This suggests that synaptic vesicle recycling may be increased in mice prone to drinking greater amounts of ethanol. Conversely, low drinking mice had higher *Bdnf* (NM_001037955) expression. *Bdnf* may play a role to increase synaptogenesis in these studies as it has been implicated in plasticity from multiple drugs of abuse [34], [35]. Moreover, BDNF has been demonstrated to increase expression of genes correlated with synaptic vesicle release, suggesting a possible causal relationship with the synaptic vesicle-related genes mentioned above.

**Table 3:** Genes from nucleus accumbens involved in synaptic vesicle formation and recycling.

Gene Name	Gene Symbol	Probeset ID	R value	q value
adaptor protein complex AP-2, alpha 1 subunit	*Ap2a1*	1460724_at	0.484	2.55E-03
adaptor protein complex AP-2, alpha 2 subunit	*Ap2a2*	1452490_a_at	−0.485	2.45E-03
**adaptor protein complex AP-2, mu1**	***Ap2m1***	1450894_a_at	0.634	1.35E-05
brain derived neurotrophic factor	*Bdnf*	1422168_a_at	−0.470	3.62E-03
**dynamin 1**	***Dnm1***	1460365_a_at	0.541	4.38E-04
dynamin 1-like	*Dnm1l*	1428087_at	−0.447	6.21E-03
protein kinase, AMP-activated, beta 1 non-catalytic subunit	*Prkab1*	1452457_a_at	−0.455	5.27E-03
Rab acceptor 1 (prenylated)	*Rabac1*	1427773_a_at	−0.525	7.57E-04
**RAB3A, member RAS oncogene family**	***Rab3a***	1422589_at	0.588	8.14E-05
secretory carrier membrane protein 1	*Scamp1*	1426775_s_at	0.450	5.80E-03
SH3-domain GRB2-like 2	*Sh3gl2*	1418792_at	−0.451	5.74E-03
**SH3-domain GRB2-like B1 (endophilin)**	***Sh3glb1***	1418011_a_at	0.612	3.23E-05
solute carrier family 1 (neuronal/epithelial high affinity glutamate transporter), member 1	*Slc1a1*	1448299_at	0.461	4.48E-03
solute carrier family 2 (facilitated glucose transporter), member 1	*Slc2a1*	1426600_at	0.458	4.82E-03
**synaptophysin**	***Syp***	1448280_at	0.573	1.48E-04
synaptotagmin II	*Syt2*	1420418_at	−0.515	9.91E-04
syntaxin 6	*Stx6*	1431646_a_at	−0.507	1.29E-03
**vesicle-associated membrane protein 3**	***Vamp3***	1437708_x_at	0.552	3.06E-04
vesicle-associated membrane protein 4	*Vamp4*	1422896_at	0.459	4.76E-03

Genes in bold were also identified by principal component analysis.

The gene lists generated from PC analysis were similar in biological function. Overrepresented Gene Ontology categories included the synaptic vesicle, chromatin modification, histone methylation, Na^+^K^+^ ATPase activity and protein kinase activity (Table S5). Many of the genes highlighted in the chromatin modification and synaptic vesicle formation and recycling networks described above were present in the principal component analysis (highlighted in bold in Table 2).

#### Prefrontal Cortex

Primary analyses of gene transcripts differentially regulated by ethanol drinking in the prefrontal cortex yielded 850 transcripts by RMA summarization, using a false discovery rate of 1% (Table S2). The gene list was entered into EASE and Bibliosphere analysis to identify over-represented functional categories as compared to all the transcripts on the Mouse430Av2 chips. The following categories were statistically over-represented at p<0.05 in both analyses (see Table S4): mitochondrial inner membrane, oxidoreductase activity, cell projection and regulation of cell shape. The top canonical pathways identified by Ingenuity Pathway Analysis mirrored some results from EASE and Bibliosphere (mitochondrial dysfunction and ubiquinone biosynthesis) and identified involvement of other signaling pathways: IL2, Pten, Jak/Stat and glucocorticoid receptor signaling.

**Figure 3:**
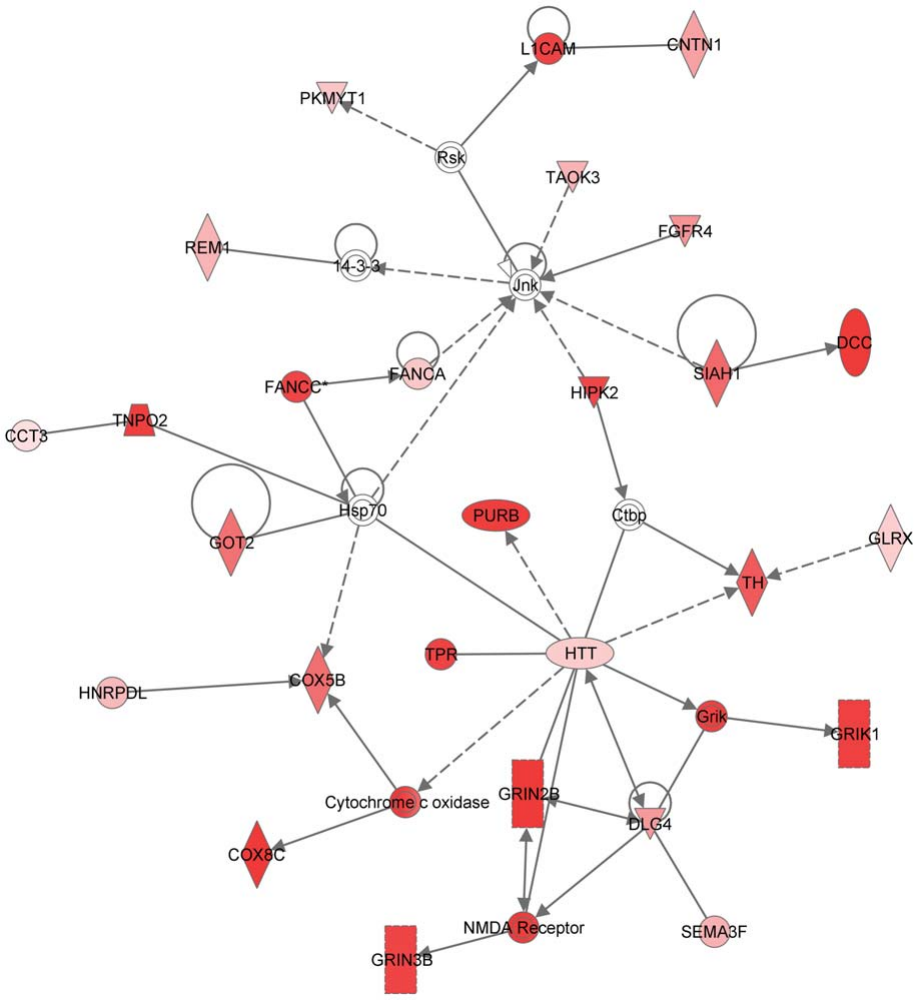
Network of genes involved in glutamate signaling in the prefrontal cortex of ethanol drinking mice. Network of genes involved in glutamate signaling generated through the use of Ingenuity Pathways Analysis (www.ingenuity.com). Genes significantly correlated to ethanol drinking are colored pink to red based on significance.

Ingenuity network analysis identified potential involvement of PFC glutamate receptor signaling (Figure 3) in the variation of ethanol drinking behaviors. This network contained several ionotropic glutamate receptor subunits, NMDA receptor subunits 2B and 3B (*Grin2b* (NM_008171), *Grin3b* (NM_028388)) and the kainite receptor (*Grik1* (NM_010348)), as well as genes that bind (*Htt* (NM_010414)) or are regulated by glutamate receptors (*Dlg4* (NM_001109752) aka *Psd95*). The NR2b subunit of the NMDA receptor was positively correlated to ethanol drinking with the lowest drinking mice having lower expression (Figure 3), while the NR3b subunit and kainate receptor (*Grik1*) were correlated negatively to ethanol drinking. Tyrosine hydroxylase, the rate-limiting enzyme in catecholamine synthesis involved in the conversion of tyrosine to dopamine, was also positively correlated to ethanol drinking (Figure 3).

Functional overrepresentation analysis of the PFC gene list derived from principal component derivation of the behavioral data revealed biological categories related to mitochondria such as electron transport, respiratory chain and mitochondrial dysfunction (Table S5) that overlapped with the averaged drinking data analysis. Two glutamate receptor subunits, *Grin2b* and *Grik1*, were also present in the PC analysis. Stress activated protein kinase signaling and retinoic acid signaling were over-represented in the principal component analysis, but not in the average drinking correlation analysis.

#### Ventral Midbrain Region

In the VMB, 559 transcripts were significantly correlated to ethanol drinking intake, using a false discovery rate of 1%. Gene Ontology analysis revealed only a few significant categories (Table S3) that were surprisingly cohesive (locomotor behavior, cell adhesion, cell projection and basolateral plasma membrane), suggesting cell migration and chemotaxis may be affected in the VMB. Corresponding analysis using Bibliosphere and the canonical pathways in Ingenuity identified many of the same categories (Table S3). Gene networks identified by Ingenuity Pathway Analysis did not reveal additional conserved biological functions for the ventral midbrain region.

Despite having a slightly lower percentage of genes overlapping with data from the average drinking analysis, the VMB gene list correlating to principal component analysis of the behavioral data had similar functional categories identified by Gene Ontology analysis (glycosaminoglycan degradation, locomotor behavior, and toll-like receptor signal; see Table S4).

### Characterization of select genes

**Figure 4:**
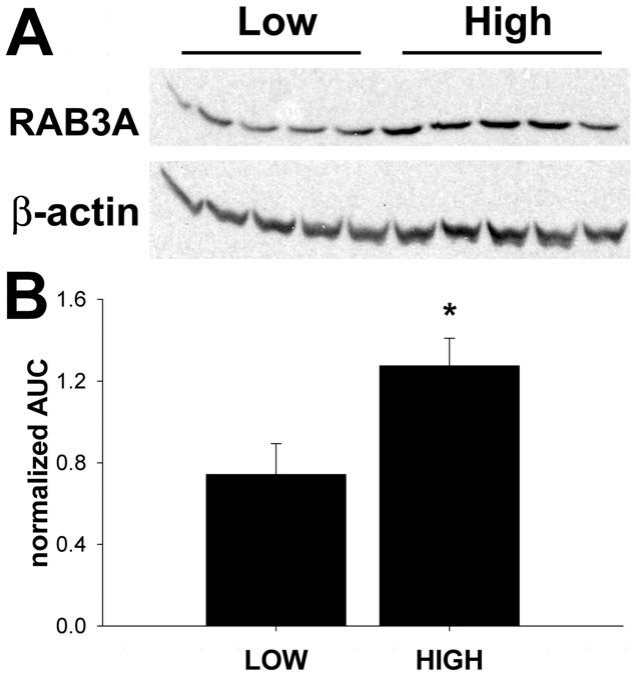
RAB3A expression in high and low drinking mice. **A**. Western blot of nucleus accumbens total protein probed with RAB3A and beta-actin. **B**. Quantitation of western blot analysis, area under the curve (AUC) RAB3A expression normalized to total beta-actin. LOW <2 g/kg EtOH, HIGH >7 g/kg EtOH. * p<0.05, by t-test.

We used Western blot analysis to further confirm the microarray results of select genes. RAB3A (NM_009001) was chosen for its documented role in synaptic vesicle trafficking. In a separate cohort of mice (Exp. 2), RAB3A expression was determined in high (n = 5, ethanol intake >7-g/kg) and low (n = 5, ethanol intake <2-g/kg) drinking mice (Figure 4). RAB3A expression was significantly lower in mice consuming less than 2-g/kg ethanol than in mice consuming more than 7-g/kg ethanol (p<0.05, T-test). Western blot analysis showed a 1.7 fold greater level of RAB3A expression in high drinking mice, similar to what was seen with mRNA expression changes in the microarray results. Additional protein studies were conducted for BDNF (Bdnf; NM_007540)and dynamin (Dnm1; NM_010065). These showed trends but did not reach statistical significance (p = 0.3 and p = 0.2, respectively), as might be expected from the small magnitude changes in mRNA abundance seen with microarrays. Rather than study larger numbers of animals for protein quantitation, we further validated our array results through bioinformatics and behavioral pharmacology studies as outlined below.

### Associations with genetic ethanol drinking phenotypes

Extensive prior microarray studies have been done comparing basal brain gene expression across multiple mouse strains with divergent ethanol drinking phenotypes. A large meta-analysis of this data identified gene expression correlated with ethanol drinking behavior across these multiple genetic models [27]. We predicted that a subset of genes having correlation with individual drinking behavior within a single inbred strain would overlap with the genetically derived gene sets associated with drinking behavior. Out of 889 significantly regulated transcripts in the NAc, 202 transcripts (p<10^−34^, Chi-square analysis) were also identified in the meta-analysis (see Figure S2b). Functional categories of these genes remained similar to our original analysis. PI3K/Akt signaling, protein ubiquitination and genes involved in synaptic vesicles were still highly represented. One of these genes, syntaxin-binding protein 1 (*Stxbp1* (NM_009295)) has been previously identified as a putative candidate for an ethanol-drinking locus on Chromosome 2 [12]. In the PFC, 168 genes out of 850 (p<10^−18^, Chi-square) were also identified by the meta-analysis (see Figure S2c). Mitochondrial dysfunction and PTEN signaling remained top biological functions. However, genes involved in glutamate receptor signaling were not represented on this list since this category was not enriched in the meta-analysis. Genes involved in retinoic acid signaling were over-represented in our principal component analysis and were also in the meta-analysis dataset. Retinoic acid signaling plays a role in the differentiation and function of dopaminergic pathways [36]. In the VMB, 108 genes out of 435 (p<10^−4^, Chi-square) were in common with the meta-analysis results (Figure S2d). Glycosaminoglycan degradation and cell movement were again identified as top biological functions. The highly significant overlap between our gene list and those of the meta-analysis across mouse lines genetically selected for differences in ethanol intake as well as the degree of overlap between functional gene categories in these two independent studies, both serve to validate our microarray data in terms of relevance to ethanol consumption.

### Histone Deacetylase Inhibition and Ethanol Drinking

**Figure 5:**
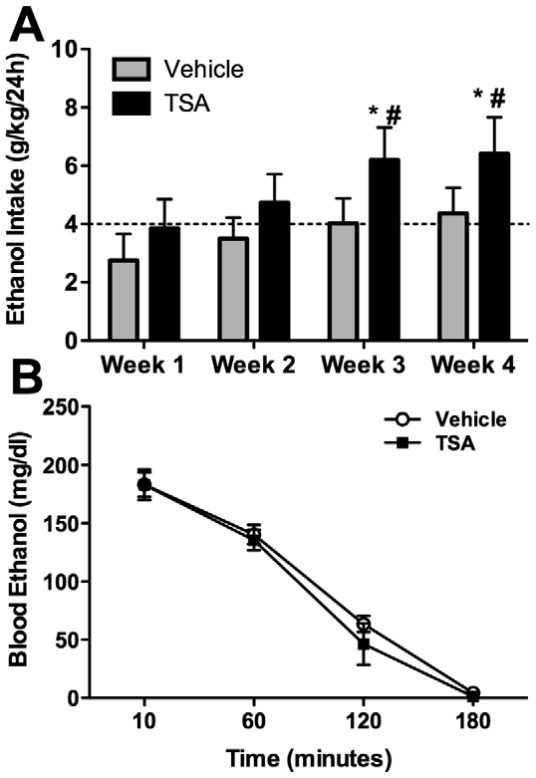
Histone deacetylase activity inhibition increases ethanol intake. **A.** Average weekly ethanol intake of C57BL/6NCrl mice after i.p. treatment with 2 mg/kg trichostatin A (TSA) or vehicle (n = 9) for 5 days. Both groups had identical baseline average ethanol intake (dotted line) prior to treatment with TSA. TSA caused ethanol intake to increase significantly by weeks 3–4 post-treatment (*p<0.001 vs. baseline; #p<0.02 vs. vehicle at same time points). **B.** Blood ethanol concentration in TSA and vehicle treated mice following a 2-mg/kg ethanol injection (i.p.).

The bioinformatics analysis of NAc genes correlating with ethanol intake showed an over-representation for genes involved in chromatin remodeling, particularly histone acetylation. Such epigenetic modifications have been shown to play a role in responses to other drugs of abuse [37], [38]. To perform a functional validation of our microarray data regarding the role of chromatin modification genes in ethanol drinking, Trichostatin A (TSA), an inhibitor of class I (HDAC isoforms 1, 2, 3, 8 and 11) and class II HDACs (isoforms 4, 5, 6, 7, 9 and 10) [39], [40], was examined to determine effects on ethanol drinking. Similar doses of TSA have been shown to increase histone acetylation in the brain, rescue memory consolidation and increase cFos and cJun mRNA expression [41], [42]. We hypothesized that if chromatin acetylation events were indeed involved in the drinking phenotype, administration of an HDAC inhibitor would alter ethanol intake/preference and potentially reduce individual variation in drinking behavior. Following baseline ethanol drinking for seven days, TSA (2 mg/kg, i.p.) was administered for five consecutive days. Ethanol was freely available 24 hours/day during and for 4 weeks following TSA administration. Both TSA and vehicle-treated groups showed decreased ethanol intake on treatment days, likely due to the acute stress of injections (not shown). Following the treatment period, TSA-treated animals showed significantly increased ethanol drinking over baseline intake at three and four weeks following administration (1-way repeated measures ANOVA with Sheffe post-hoc, p<0.01) while vehicle treated animals showed no significant change (Figure 5A). Vehicle treated animals showed a gradual return to baseline drinking over the four weeks following injections. Two-way repeated measures ANOVA using weekly ethanol intake after treatment as the dependent variable revealed a main effect of time (F(3, 72) = 9.03, p<0.0001). Treatment and treatment x time interactions did not show significant effects likely due to gradual onset of the TSA response and the slight time-dependent changes in the vehicle-treated animals. However, post-hoc analyses indicated that vehicle and TSA-treated group ethanol intakes were significantly different from one another at week 3 and week 4 (Sheffe, p<0.02). There was no effect of TSA treatment on total fluid consumed over the course of the experiment (data not shown) and, in a separate experiment, TSA treatment had no effect on the kinetics of ethanol metabolism following an i.p. injection of 2-g/kg ethanol (Figure 5B). Western blot analysis for histone H4 hyperacetylation confirmed elevated H4 acetylation levels 24 hours after a single 2-mg/kg TSA i.p. injection (Figure S3).

We hypothesized that TSA treatment might also reduce the variability of ethanol intake in individual mice as well as alter overall ethanol intake. However, the relatively small sample size for this experiment (n = 9 for each treatment group) did not allow a definitive conclusion as to whether TSA affected the overall distribution of drinking values, since most animals showed increased drinking following TSA (not shown).

## Discussion

Our studies here showed that C57BL/6NCrl mice express a striking degree of stable, inter-individual variation in ethanol drinking behavior with greater than 10-fold differences within a drinking session. We suspect these differences were generated by subtle environmental differences such as rearing behaviors [43], [44], intrauterine position, social interactions or stress [45], [46]. Individual variation within the C57 substrains has been reported for ethanol drinking behaviors [16], [17], [47] as well as in stress responsiveness [48] that may be a contributing factor to ethanol preference [49], [50], [51]. For example, C57BL/6J mice are known to consume slightly more ethanol in a 2 bottle choice paradigm than C57BL/6NCrl [19], [52] and also display variation of individual ethanol intake (unpublished observation), albeit the range is smaller than with C57BL/6NCrl mice. C57BL/6J mice have several chromosomal regions that are duplicated in comparison to C57BL/6NCrl mice [52], and may account for the differences in overall consumption and range of individual variation between the two strains. In the C57BL/10 substrain, Little *et al*. reported that within-strain preference variation was not correlated with gender or ethanol metabolism, and could not be altered by simple environmental disturbances [18]. Gonzalez et al. have shown that C57BL6/J and C57BL/6NCrl also do not differ in ethanol metabolism rates [53]. Regardless of which environmental conditions may have contributed to variation in ethanol drinking behaviors, we hypothesized that the differences could be mediated by individual variation in basal gene expression.

The studies here employed a unique experimental design that allowed long-term measures of ethanol drinking behavior, ensured that such behavior was stable upon reinstatement, and permitted assaying gene expression differences in individual animals off ethanol. This allowed identification of expression patterns presumably “predictive” of drinking behavior rather than simply resulting from such. However, even with the current design, we cannot totally eliminate the possibility that some of our gene expression results reflect, rather than cause, individual variation in drinking behavior. Additionally, even though the behavioral data showed extinction of an ethanol deprivation effect after several cycles of withdrawal and reinstatement (Figure S1), we cannot exclude that some of our microarray results do indeed reflect expression changes secondary to ethanol deprivation. Perhaps most likely, there could be a complex interaction between basal, ethanol-deprivation and ethanol-responsive gene expression patterns underlying these microarray results and perpetuating the long-term drinking patterns seen in these animals. However, as discussed below, the highly significant overlap of our expression gene sets with data from a meta-analysis on basal gene expression correlating with ethanol consumption [27], strongly suggests that our results largely represent basal individual variation in gene expression that influenced drinking behavior.

The current studies showed a potential role for epigenetic regulation of ethanol preference in B6 mice. In the NAc, genes with chromatin remodeling Gene Ontology function or classified in the HDAC complex had differential expression between high and low ethanol-drinking animals (see Table 2). Intriguingly, genes involved in both histone modifications as well as genes involved in DNA methylation events were significantly altered in the nucleus accumbens but not in other brain regions assayed. Thus, our genomic findings suggest an extensive and complex representation of chromatin modification gene networks as contributing to variation in ethanol intake specifically in the nucleus accumbens.

Inhibiting HDAC activity with TSA injections increased ethanol intake above baseline and vehicle-treated levels, supporting a role for chromatin modifications in the modulation of ethanol preference. The complex changes in chromatin modification gene expression made it difficult to predict how directly altering histone acetylation might affect drinking behavior. We suggest, however, that any TSA-induced change in ethanol drinking across individuals or as a population is supportive of our hypothesis regarding a role of chromatin modification in driving individual variation in ethanol intake. This data is the first to show modulation of drinking behavior by altering chromatin acetylation. However, evidence of ethanol-induced chromatin remodeling has been reported in hepatocytes [54] and in mouse brain [55]. In cultured cortical neurons, ethanol increases NR2B transcription possibly through epigenetic modifications such as methylation of CpG islands [56]. Acute ethanol increases histone H3 and H4 acetylation and decreases HDAC activity in amygdala, while ethanol withdrawal produces the opposite response with decreased histone acetylation [57]. Social stress induces histone H3 demethylation at certain *Bdnf* promoters, leading to decreased *Bdnf* transcription [58]. Together, these studies demonstrate that environmental factors such as social stress or drug taking can modify chromatin and support a role for chromatin remodeling in the formation of stable neuronal adaptations underlying individual differences in drinking behavior.

Our bioinformatics analysis of gene expression correlating with ethanol drinking also identified gene networks involved in synaptic vesicle biogenesis and recycling (Table 3). Many of these genes have previously been implicated as playing a role in ethanol drinking or acute response to ethanol. For example, syntaxin-binding protein, STXBP1, anchors synaptic vesicles to the plasma membrane and was positively correlated to ethanol drinking in our studies. *Stxbp1* was previously identified as a candidate gene for a mouse Chr2 ethanol preference locus [12]. RAB3A, a small GTPase associated with synaptic vesicle trafficking and neurotransmitter release [59], was positively correlated to ethanol intake and protein expression was 1.7 fold higher in heavy drinking mice. This gene may play a role in sensitivity to the acute ataxic and sedative effects of ethanol in *C. elegans* and mice [60].

We also identified an inverse correlation between *Bdnf* mRNA levels and individual ethanol consumption. BDNF regulates multiple synaptic vesicle-related proteins, including several listed in Table 3, such as synaptotagmin, synaptophysin [61], AP2 complexes [62], and STXBP1 [63]. BDNF has been implicated in neuroplasticity from multiple drugs of abuse [34], [35]. In clinical studies, peripheral BDNF is lower in dependent alcoholics and patients with a positive family history of dependence as compared to normal controls and dependent patients with a negative family history [64]. McGough et al. [65] also showed that *Bdnf* under-expression in *Bdnf*+/- mice caused increased ethanol consumption, consistent with *Bdnf* mRNA expression observed in the current study, where *Bdnf* is lowest in mice with the highest ethanol intake. We do not believe, therefore, that *Bdnf* expression levels seen in our studies were secondary to ethanol exposure itself. In support of this, we and other investigators have shown that acute ethanol injection (2 g/kg i.p.) in B6 or D2 mice increases *Bdnf* expression and that after 4 weeks of 2-bottle choice ethanol drinking, *Bdnf* is increased in the dorsal striatum versus non-ethanol controls [20], [65]. Thus, we suggest that lower *Bdnf* expression in the low drinking mice was possibly a causal factor in individual drinking behavior variance, rather than secondary to drinking behavior itself. We cannot currently exclude the possibility that ethanol drinking followed by withdrawal (4 days) caused the correlated changes in *Bdnf* expression. Together, these findings on *Bdnf* and synaptic vesicle-related gene expression are strong evidence supporting an important link between regulation of synaptic vesicles and individual variation in ethanol intake.

In the present study, many of the most robust gene expression changes were found in the nucleus accumbens and prefrontal cortex. Brain regional differential gene expression is not surprising considering the proposed different roles of each region in ethanol responses [66]. Furthermore, we have seen such inter-region diversity with our prior studies on acute ethanol– such as *Bdnf* expression only being regulated in nucleus accumbens [20]. The regional differences are potentially functionally significant. For example, glutamate signaling, the major excitatory feedback to the ventral tegmental area, was altered in prefrontal cortex. The finding of expression differences related to potential epigenetic regulation events only in nucleus accumbens, is particularly intriguing given the known role of that region in drug reward.

Importantly, we found significant overlap between our gene lists and a previously published meta-analysis of basal brain gene expression across mouse strains with differing ethanol preference [27]. Several functional categories potentially involved in drinking phenotypes were also over-represented in both studies, including PI3K/Akt and PTEN signaling, protein ubiquitination and mitochondrial dysfunction. These functional categories together suggest a role for cell survival pathways, altered energy metabolism or potential neuronal toxicity due to ethanol consumption. However, animals from the meta-analysis never consumed ethanol. Therefore it is possible that animals with a proclivity to drink ethanol may have altered signaling in these pathways prior to drinking.

In conclusion, the current experiments have described persistent inter-individual variation of ethanol drinking behaviors in B6 mice and, more importantly, they define gene expression networks that may underlie these individual differences. This study utilizes variation within an inbred strain to minimize genetic influences, isolating changes in gene expression due specifically to environmental factors. These experiments have identified several gene networks previously implicated in responses to ethanol in the NAc and PFC: glutamate signaling, BDNF and genes involved in synaptic vesicle function. Perhaps most importantly, our expression studies and behavioral analysis following histone deacetylase inhibition implicate epigenetic factors involving chromatin acetylation and/or methylation as contributing to environmental modulation of ethanol intake. Defining specific gene networks targeted by these epigenetic modifications is an important goal of ongoing studies. The novel findings presented here could contribute to understanding mechanisms involved in individual risk for alcohol abuse and alcoholism in humans. Future work will focus on characterizing the genesis and implications of gene network alterations and epigenetic modifications associated with variation in ethanol drinking.

## Supporting Information

Figure S1 **Average Ethanol Intake Over 16 Days Of Access.** Ethanol intake was significantly increased following repeated ethanol deprivations (**p<0.001 day 4 vs. day 9, *p<0.01 day 4 vs. day 17, Bonferroni Multiple Comparison test). Ethanol consumption did not differ from baseline after the third deprivation (p>0.05, day 4 vs. day 25).
(TIF)

Figure S2 **Genes Differentially Regulated In Ethanol Drinking Mice.** **A**. Venn diagram overlapping and non-overlapping genes in each brain region significantly correlated to drinking at FDR<0.01. Region-specific expression patterns are represented as shaded circles (nucleus accumbens (NAc), dark; prefrontal cortex (PFC), open; ventral midbrain (VMB), light). **B**–**D.** Hierarchical clustering of transcripts significantly correlated to ethanol drinking in the NAc (**B**), PFC (**C**) and VMB (**D**). Genes that overlap with the meta-analysis are labeled in blue. Genes that overlap with the principal component analysis are labeled in orange. Red color indicates higher relative expression and green indicates lower expression. Columns are arranged according to drinking behavior averaged over the last 8 days of intake, with low drinking mice on the left, progressing to higher drinking mice on the right.
(TIF)

Figure S3 **Western Blot Analysis Of TSA Effects On Histone H4 Acetylation.** Western blotting for acetyl-Histone-H4 was used to verify CNS activity of i.p. TSA. C57BL/6NCrl mice (n = 12) received a single i.p. injection of 2 mg/kg TSA or vehicle. After 24 hours, nucleus accumbens was dissected for Western blotting for acetyl-histone H4 (upper panel) or GAPDH as a loading control (lower panel). Results verify increased H4 acetylation in NAc after TSA treatment.
(TIF)

Table S1 Average Ethanol Intake and Preference for six C57BL/6NCrl litters. Average ethanol intake and preference for six separate litters of male mice (n = 4−5/litter) was calculated over 14 days of 24 h access to 2-bottle choice ethanol. There was no significant difference in intake or preference as reported in Results.
(XLSX)

Table S2 Results from Correlation with Average Drinking. Data are gene lists and statistics for gene expression correlating with individual average ethanol intake from the last 8 days of ethanol access following a third round of ethanol deprivation. Data are presented for each brain region separately.
(XLSX)

Table S3 Gene Lists from Principal Component Analysis. Data are gene lists and statistics for gene expression correlating with ethanol intake data subjected to principal component analysis as in Methods following a third round of ethanol deprivation. Data are presented for each brain region separately. Categories in bold were also significant in the averaged drinking analysis (Table S2).
(XLSX)

Table S4 Over-Represented Gene Categories From Gene Expression Significantly Correlated To Average Ethanol Drinking. Gene data from Table S2 was analyzed by functional over-representation analysis versus Gene Ontology categories. Categories in bold were also significant in the Bibliosphere analysis.
(XLSX)

Table S5 Gene Categories Significantly Over-Represented From Principal Component Analysis In Ethanol Drinking C57BL/6NCrl Mice. Gene set data from Table S3 was analyzed by functional over-representation analysis versus Gene Ontology categories. Gene categories in bold were also significantly over-represented in the correlation analysis (Table S4).
(XLSX)
