# Supplementary figures and images for: Genomic Analysis of Individual Differences in Ethanol Drinking: Evidence for Non-Genetic Factors in C57BL/6 Mice
Source: PLoS One. 2011 Jun 16;6(6):e21100. doi: 10.1371/journal.pone.0021100 (PMC3116881; doi:10.1371/journal.pone.0021100)

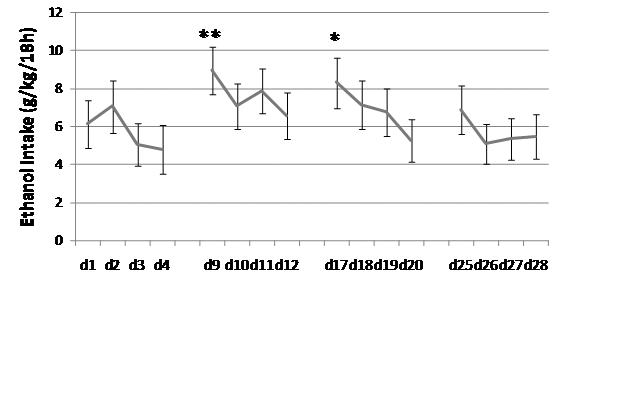

Supplement: Figure S1 — Average Ethanol Intake Over 16 Days Of Access. Ethanol intake was significantly increased following repeated ethanol deprivations (**p<0.001 day 4 vs. day 9, *p<0.01 day 4 vs. day 17, Bonferroni Multiple Comparison test). Ethanol consumption did not differ from baseline after the third deprivation (p>0.05, day 4 vs. day 25). (TIF) [file pone.0021100.s001.tif]

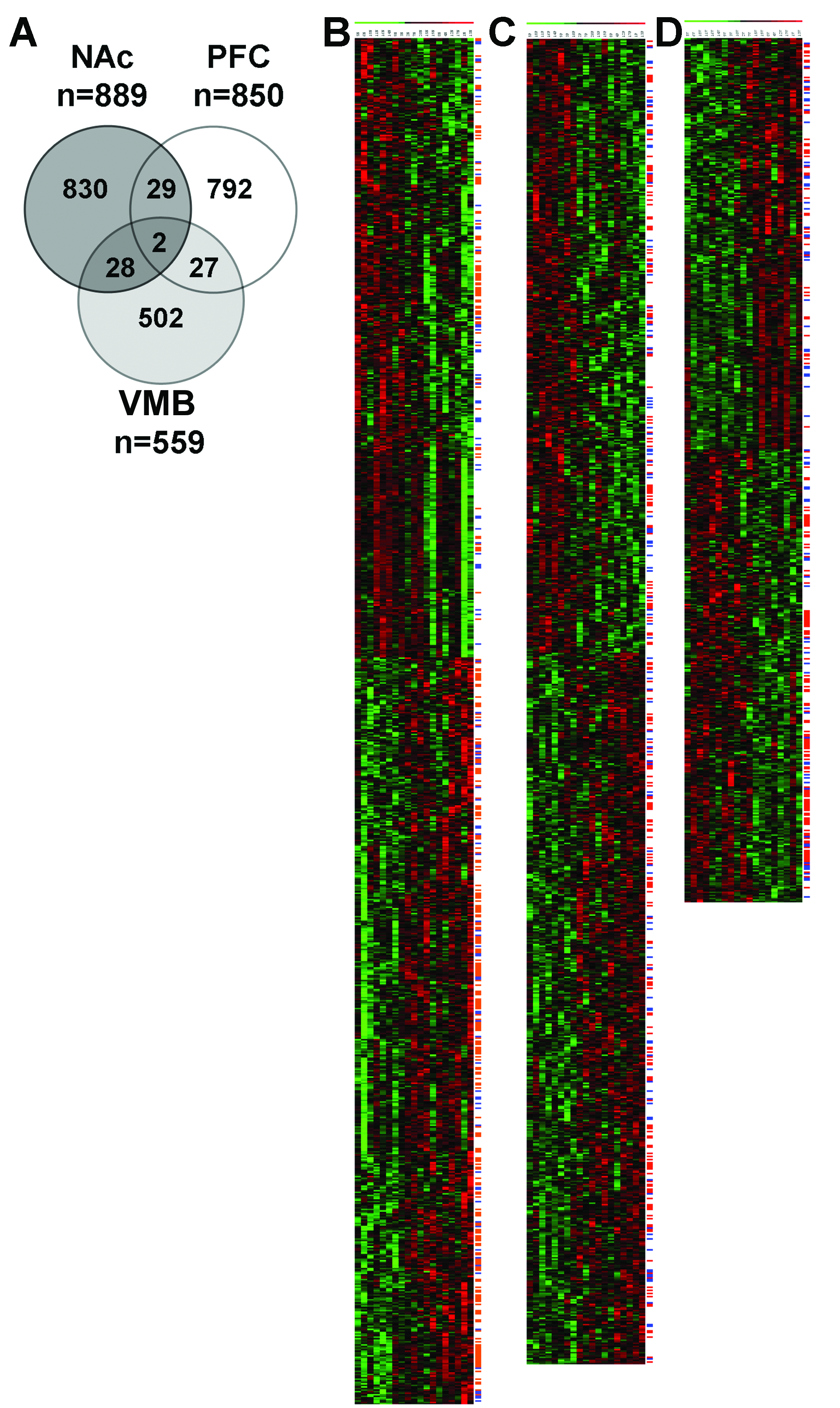

Supplement: Figure S2 — Genes Differentially Regulated In Ethanol Drinking Mice. A. Venn diagram overlapping and non-overlapping genes in each brain region significantly correlated to drinking at FDR<0.01. Region-specific expression patterns are represented as shaded circles (nucleus accumbens (NAc), dark; prefrontal cortex (PFC), open; ventral midbrain (VMB), light). B–D. Hierarchical clustering of transcripts significantly correlated to ethanol drinking in the NAc (B), PFC (C) and VMB (D). Genes that overlap with the meta-analysis are labeled in blue. Genes that overlap with the principal component analysis are labeled in orange. Red color indicates higher relative expression and green indicates lower expression. Columns are arranged according to drinking behavior averaged over the last 8 days of intake, with low drinking mice on the left, progressing to higher drinking mice on the right. (TIF) [file pone.0021100.s002.tif]

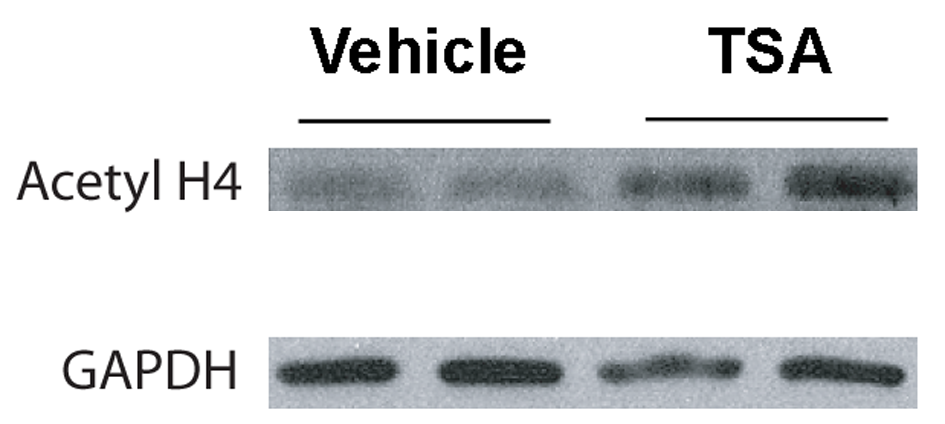

Supplement: Figure S3 — Western Blot Analysis Of TSA Effects On Histone H4 Acetylation. Western blotting for acetyl-Histone-H4 was used to verify CNS activity of i.p. TSA. C57BL/6NCrl mice (n = 12) received a single i.p. injection of 2 mg/kg TSA or vehicle. After 24 hours, nucleus accumbens was dissected for Western blotting for acetyl-histone H4 (upper panel) or GAPDH as a loading control (lower panel). Results verify increased H4 acetylation in NAc after TSA treatment. (TIF) [file pone.0021100.s003.tif]
